# Predictive Values of Preoperative Characteristics for 30-Day Mortality in Traumatic Hip Fracture Patients

**DOI:** 10.3390/jpm11050353

**Published:** 2021-04-28

**Authors:** Yang Cao, Maximilian Peter Forssten, Ahmad Mohammad Ismail, Tomas Borg, Ioannis Ioannidis, Scott Montgomery, Shahin Mohseni

**Affiliations:** 1 Clinical Epidemiology and Biostatistics, School of Medical Sciences, Örebro University, 70182 Örebro, Sweden; scott.montgomery@oru.se; 2 Unit of Integrative Epidemiology, Institute of Environmental Medicine, Karolinska Institute, 17177 Stockholm, Sweden; 3 Department of Orthopedic Surgery, Orebro University Hospital, 70185 Orebro, Sweden; maximilian.forssten@oru.se (M.P.F.); ahmad.mohammmad-ismail@oru.se (A.M.I.); tomas.borg@oru.se (T.B.); ioannis.ioannidis@oru.se (I.I.); 4 School of Medical Sciences, Orebro University, 70182 Orebro, Sweden; shahin.mohseni@oru.se; 5 Clinical Epidemiology Division, Department of Medicine, Karolinska Institutet, 17177 Stockholm, Sweden; 6 Department of Epidemiology and Public Health, University College London, London WC1E 6BT, UK; 7 Division of Trauma and Emergency Surgery, Department of Surgery, Orebro University Hospital, 70185 Orebro, Sweden

**Keywords:** hip fracture, postoperative mortality, prediction, variable importance, machine learning, neural network

## Abstract

Hip fracture patients have a high risk of mortality after surgery, with 30-day postoperative rates as high as 10%. This study aimed to explore the predictive ability of preoperative characteristics in traumatic hip fracture patients as they relate to 30-day postoperative mortality using readily available variables in clinical practice. All adult patients who underwent primary emergency hip fracture surgery in Sweden between 2008 and 2017 were included in the analysis. Associations between the possible predictors and 30-day mortality was performed using a multivariate logistic regression (LR) model; the bidirectional stepwise method was used for variable selection. An LR model and convolutional neural network (CNN) were then fitted for prediction. The relative importance of individual predictors was evaluated using the permutation importance and Gini importance. A total of 134,915 traumatic hip fracture patients were included in the study. The CNN and LR models displayed an acceptable predictive ability for predicting 30-day postoperative mortality using a test dataset, displaying an area under the ROC curve (AUC) of as high as 0.76. The variables with the highest importance in prediction were age, sex, hypertension, dementia, American Society of Anesthesiologists (ASA) classification, and the Revised Cardiac Risk Index (RCRI). Both the CNN and LR models achieved an acceptable performance in identifying patients at risk of mortality 30 days after hip fracture surgery. The most important variables for prediction, based on the variables used in the current study are age, hypertension, dementia, sex, ASA classification, and RCRI.

## 1. Introduction

Hip fractures are one of the most common type of fractures in elderly people with an incidence that has doubled in the last 20 years and is expected to keep growing [1,2,3,4,5]. In Sweden, the annual incidence of hip fractures is approximately 18,000 per year [6,7,8]. Hip fracture patients have a high risk of mortality after surgery, with 30-day postoperative rates as high as 10% [9]. Despite efforts made to mitigate this, the death rate after hip fracture surgery has remained relatively unchanged during the last decades [9].

With the goal of reducing the mortality rates in this patient population, it is vital to explore and understand the factors that potentially could predict postoperative mortality. This has previously been attempted using both logistic regression models and neural networks [10,11,12]. Neural networks require more data and variables to be more reliable, but also provide a unique combination of properties that cannot be assessed using traditional generalized linear models. On the other hand, logistic regression (LR) is a commonly used classifier in machine learning classification problems, which has been widely applied in clinical research and is familiar to the surgeons. A single-layer feed-forward neural network that uses a logistic transfer function is structurally identical to a LR model [13]. LR allows the most statistically significant inputs to be found in a simple manner that highlights important variables that may aid in the decision-making processes. Furthermore, the size and direction of the effect of each covariate can be determined in a LR model to estimate the effect of the weighted sum of the covariates, and a risk score calculated accordingly [14]. Outcome prediction models using neural networks could also be useful tools in attempting to reduce mortality after hip fracture surgery by identifying modifiable risk factors. Consequently, the current study aims to explore the predictive values of preoperative characteristics in traumatic hip fracture patients for 30-day postoperative mortality by using a convolutional neural network (CNN) and a LR model, in addition to comparing the performance of the two algorithms.

## 2. Materials and Methods

### 2.1. Study Population

The data were obtained from the Swedish National Quality Registry for Hip Fracture Patients, *Rikshöft* [15]. All adult patients ≥ 18 years old, who underwent primary emergency hip fracture surgery in Sweden between 1 January 2008 and 31 December 2017, were included in the analysis. Pathological and conservatively treated hip fractures were excluded from the original data retrieval. The dataset was cross referenced with the Swedish National Board of Health and Welfare registers using the patients’ unique social security numbers, which provided each patient’s date of death and comorbidity data. The comorbidity data was used to determine both the Charlson Comorbidity Index (CCI) and the Revised Cardiac Risk Index (RCRI) for each patient [16,17]. The RCRI was calculated based on a history of ischemic heart disease, congestive heart failure, cerebrovascular disease, renal insufficiency, and diabetes mellitus, with each variable counting as one point if present [18]. This study complies with the principles of the Declaration of Helsinki as well as the TRIPOD guidelines [19], and was approved by the Regional Ethical Review Authority of Uppsala/Orebro (reference 2019-02094).

### 2.2. Primary Outcome and Predictor Variables

The primary outcome is death within 30 days after surgery for a traumatic hip fracture. The following variables were used in the current study for prediction of the primary outcome: age, sex, fracture type, type of surgery, RCRI, CCI, American Society of Anesthesiologists (ASA) classification, and preoperative comorbidities, including peripheral vascular disease (PVD), chronic obstructive pulmonary disease (COPD), liver disease, dementia, connective tissue disease, cancer, metastatic carcinoma, myocardial infarction (MI), congestive heart failure (CHF), hypertension, arrhythmia, cerebrovascular disease (CeVD), peptic ulcer disease, diabetes mellitus, hemiplegia, and chronic kidney disease.

### 2.3. Descriptive Analysis and Association Analysis

Continuous variables were presented as a mean ± standard deviation (SD), and the ordered and nominal variables were presented as a count and percentage. The Pearson’s chi-squared test was used to test the statistical significance of differences between groups for categorical variables, while the Student’s t-test and Mann–Whitney U test were used for continuous variables. Because the rate of incomplete observations is very low (<2.0%), the listwise deletion method was used for the observations with missing values in the variables used in a specific analysis [20]. Two-tailed *p*-values < 0.05 were considered statistically significant. Associations between the predictor variables and the outcome were evaluated using a multivariate LR model. To exclude the statistically non-significant variables from the LR model, the bidirectional stepwise variable selection was used with the training dataset, with the p-value for entry being 0.05 and the *p*-value for removal being 0.10 [21].

### 2.4. Predictive Models

Two supervised machine learning (ML) algorithms, LR and CNN, were used in the current study. To reduce the overfitting of the models, we also introduced different penalties in the LR model, including L1, L2, and elastic net, as well as only included variables with a relative higher importance in both the prediction models. In the CNN model, ten layers were used: three dropout layers (one for the input layer and two for the hidden layers), two one-dimensional (1D) convolution layers, two 1D max pooling layers, one flattened layer, and two dense layers (with 1000 computation units). The rectified linear unit (ReLU) activation function was used for the convolution layers and the first dense layers, and the sigmoid activation function was used for the second dense layer. The binary cross-entropy loss function and the adaptive moment estimation (Adam) optimizer were used when compiling the model (Figure S1). Furthermore, the number of filters were changed in the conventional layers, along with the number of epochs used during model training to find an optimal CNN model for final prediction [22,23]. Optimization of the hyperparameters, including the penalty parameter for the LR model was, achieved by using a grid search method.

### 2.5. Data Augmentation and Normalization

The proportion of patients who died within 30 days postoperatively is relatively low (7.6%). This imbalance in positive and negative outcomes may significantly affect the predictive ability of the ML models; therefore, a synthetic minority oversampling technique (SMOTE) was performed to artificially augment the proportion of patients who died. SMOTE generates a synthetic instance by interpolating the *m* instances (for a given integer value *m*) of the minority class that lies close enough to each other to achieve the desired ratio between the majority and minority groups [24]. Accordingly, a SMOTE dataset with a 1:1 ratio between the living and dead patients was generated and used for machine learning.

Because feature/variable scaling or normalization is crucial when working with mixed (discrete/continuous) variables in machine learning, the categorical variables were converted into dummy variables with values of 0 and 1, and the continuous variables were standardized to have a mean of 0 and a standard deviation of 1 before they entered the CNN and LR models.

### 2.6. Model Training and Test

Randomly selected data (80%) was used as training dataset to train the CNN and LR models.

During model training stage, K-fold cross-validation technique was used [25]. The training dataset was randomly split into 5 equal partitions, which instantiated 5 identical model building and validation processes. Both the CNN and LR models were built on 4 partitions while the predictive ability was evaluated using the remaining partition. The predictive ability of the models for the training dataset was calculated as the average predictive ability over the 5 validations. The remaining 20% of the data was used as a test dataset to provide an unbiased evaluation of the final models’ fit on the training dataset (Figure S2). We also tried different partitions of the training and test datasets, i.e., 85%:15% and 90%:10%, to examine the performance of the CNN. Validation of the models for the test dataset was presented using the calibration plot with the true probability of the 30-day mortality and the predicted probability.

### 2.7. Model Performance Metrics

Given that our prediction task was a binary classification question, i.e., whether the patient would die or survive, we first used threshold-dependent metrics, including overall accuracy, sensitivity, specificity, to evaluate the performance of the CNN and LR models. Terminology and derivations of the metrics have been previously published [26]. To use the threshold-dependent performance metrics, model-predicted probabilities of presenting outcomes were transformed into binary (survival or dead) predictions by selecting a threshold probability *τ*. If a model-predicted a probability above *τ*, the prediction was classified as death, and below *τ* was classified as survival, which may be expressed as [27]:

(1) $${\hat{p}}_{i}=\{\begin{array}{cc} 1\;\left(death\right), & p_{i}>\tau \\ 0\;\left(survival\right), & p_{i}\leq \tau \end{array}$$

where ${\hat{p}}_{i}$ is a binary prediction, and *p_i_* is a model-predicted probability of death in Equation (1). For binary predictions, when the threshold *τ* is defined, a confusion matrix can be assembled, representing the joint distribution of prediction-observation pairs, from which all the threshold-dependent metrics are calculated. Because every threshold results in a different confusion matrix and consequently different threshold-dependent metrics, the change of specificity and sensitivity with *τ* increasing from 0 to 1 was presented using the receiver operating characteristic (ROC) curve. In the current study, we reported the accuracy, specificity, and sensitivity at the *τ* that maximized the Youden index (or sensitivity + specificity − 1), which emphasizes both sensitivity and specificity and is a commonly used index for identifying the optimal threshold *τ* [28].

On the other hand, because the area under the ROC curve (AUC), a threshold-independent metric, has been widely adopted by the medical society as a global measure of the predictive ability of the models [29], which avoids the potential arbitrariness associated with the selection of the threshold and is also prevalence-independent [30], we reported AUC values of the models based on the model-predicted probabilities. The boundaries for acceptable, good, and great predictive models were defined as an AUC value greater than 0.7, 0.8, and 0.9, respectively [31].

### 2.8. Variable Importance

Predictor variables’ importance was evaluated for LR models (with all the variables and the selected variables by the stepwise method) using both the LR and random forest (RF) algorithms and presented as the permutation importance (PI) and the Gini importance (GI), respectively. PI was measured by looking at how much a predefined performance metric (in our study is the sum of sensitivity and specificity) decreases when the information of a specific variable is not available. It was calculated for each variable in a model. To mask the information of a variable during validation, instead of removing the variable from the dataset, the PI method replaces it with random noise from other participants by shuffling the values of the variable. The relative importance of a variable was calculated as the accuracy decrease of the variable relative to the range of the accuracy decreases of all the variables [32]. In random forest models, the GI was calculated as the sum over the number of splits (across all trees) that include the variable, proportionally to the number of samples in each split. The GI indicates how often a particular variable was selected for a split and how large its overall discriminative value was for the classification problem under study [33].

### 2.9. Software and Hardware Used

The descriptive and ML analyses were conducted in Stata 16.1 (StataCorp LLC, College Station, TX, USA). The creation of the ML models using CNN and LR algorithms, the calculation of each model’s predictive ability, along with the PI and GI indices was performed in Python 3.6 (Python Software Foundation, https://www.python.org/, accessed on 26 April 2021) using the Keras 2.4.0, Scikit-learn 0.23 and ELI5 0.10.1 packages. All the computation was completed on a computer with 64-bit Windows 7 Enterprise operating system (Service Pack 1), Intel ^®^ Core TM i5-4210U CPU of 2.40 GHz, and 16.0 GB installed random access memory.

## 3. Results

### 3.1. Characteristics of the Patients

In total, 134,915 traumatic hip fracture cases were included in the study of whom 10,208 (7.6%) died within the 30-day postoperative period. The patients who died were on average older and more often male. There was a statistically significant difference in all characteristics and comorbidities except for connective tissue disease and hemiplegia (Table 1).

### 3.2. Associations between 30-Day Mortality and the Predictor Variables

The full LR model including all the variables indicates that age, male sex, PVD, COPD, liver disease, dementia, cancer, metastatic carcinoma, ASA classification, fracture type, CCI, hypertension, and arrhythmia are significantly associated with 30-day postoperative mortality (Table 2).

The stepwise LR model indicates that RCRI, age, male sex, PVD, COPD, liver disease, dementia, arrhythmia, cancer, metastatic carcinoma, ASA classification, fracture type, surgery type, diabetes, CeVD, hemiplegia, CHF, and hypertension are statistically significantly associated with 30-day postoperative mortality (Table 3).

### 3.3. Predictive Ability of the ML Models

**Figure 1:**
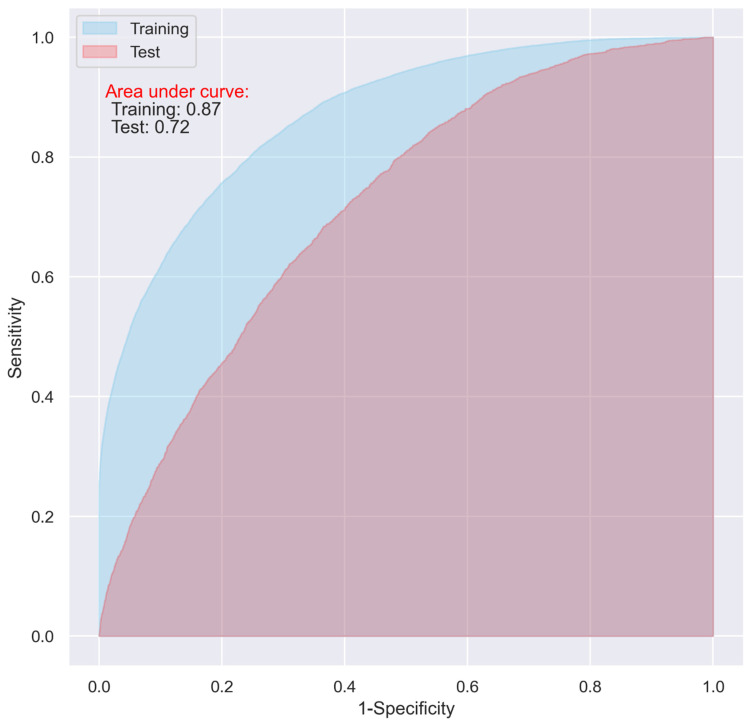
ROC of the CNN model including all the variables.

**Figure 2:**
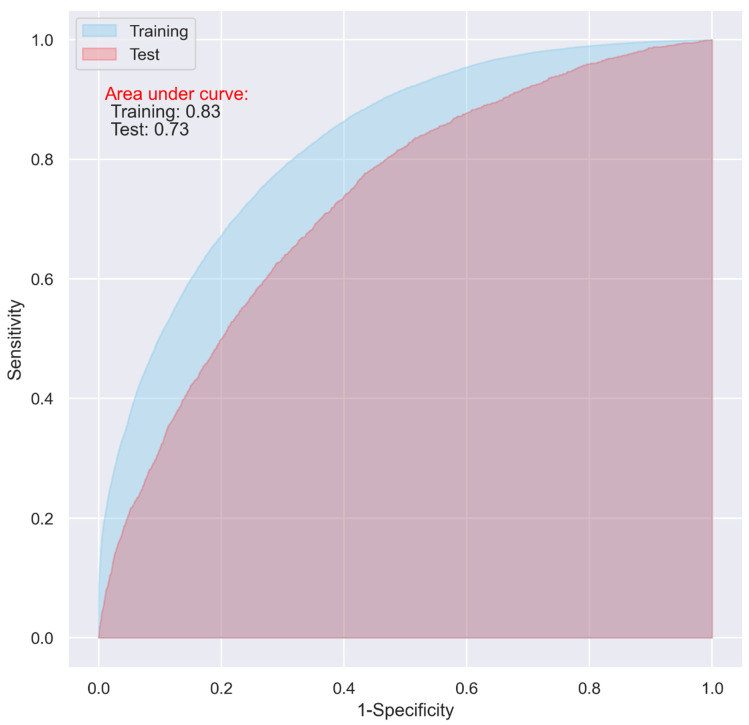
ROC of the LR model including all the variables without penalty.

While the CNN and LR models including all the predictive variables displayed a good predictive ability on the training dataset (AUC = 0.87 and 0.83, respectively), they only demonstrated an acceptable predictive ability on the test dataset for predicting 30-day postoperative mortality (AUC = 0.72 and 0.73 respectively) (Figure 1 and Figure 2).

Compared to the CNN model, the LR model exhibited a relatively higher sensitivity (0.57 vs. 0.52), and a comparable specificity (0.75 vs. 0.76) and accuracy (0.73 vs. 0.74) in the test (Table 4).

Compared to the 80%:20% partition of the training and test datasets, there was no significant change found in the results of the 85%:15% partition (AUC = 0.89 and 0.71 for the training and test datasets, respectively), and the performance of the CNN was even slightly worse for the 90%:10% partition (AUC = 0.88 and 0.69 for the training and test datasets, respectively) (Table 4).

The CNN model’s predictive ability increases on the training dataset with an increasing number of filters and epochs. However, the predictive ability was only constant or even decreased on the test dataset. When we used 50 filters and 5 epochs in the CNN model, the greatest predictive ability in terms of AUC was observed for the test dataset (Figures S3 and S4).

The overfitting issue presented in Figure 1 and Figure 2 could be alleviated with the hyperparameter optimization in the models using all the variables, however, the performance of the models on the test dataset did not show obvious improvement, which fluctuated between approximately 0.70 and 0.74.

We also evaluated the RF model with all the variables and optimized hyperparameters, which did not show any superiority over the CNN and LR models (Table 4).

### 3.4. Importance of the Predictor Variables

**Figure 3:**
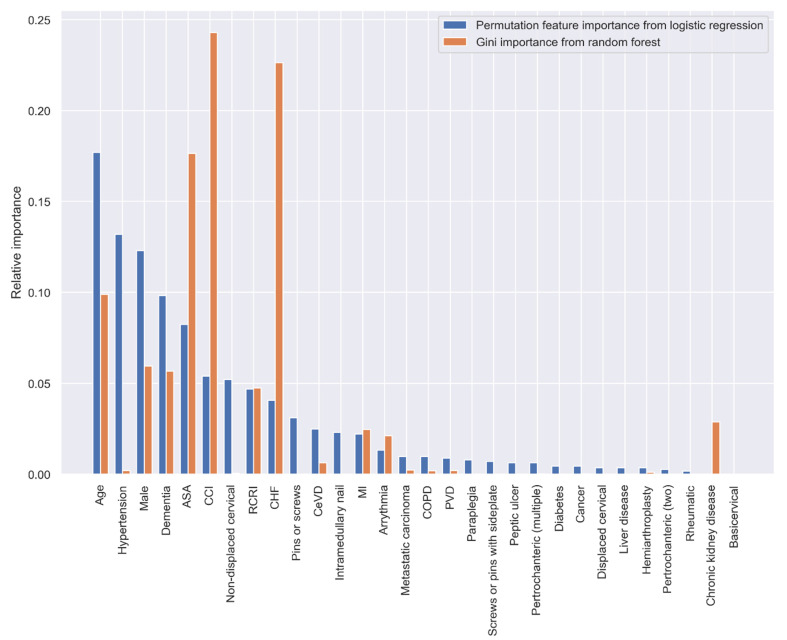
Relative variable importance in prediction 30-day mortality (all variables).

Both the PI and GI methods presented similar results regarding the importance of the variables in predicting 30-day postoperative mortality, however, the orders of the most important variables were different. When using all the predictive variables, age, hypertension, sex, dementia, and ASA classification were the most important variables according to the PI method; CCI, CHF, ASA classification, age, and sex had the highest predictive ability based on the GI method (Figure 3).

**Figure 4:**
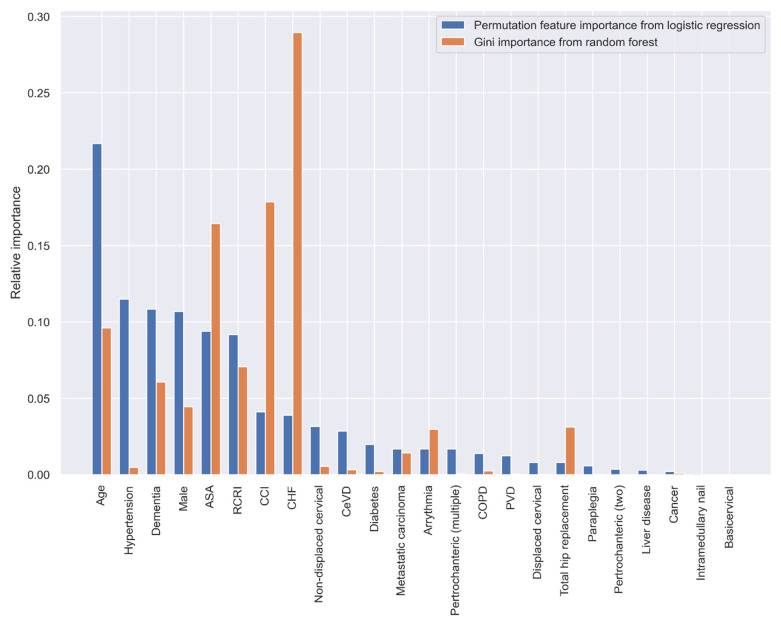
Relative variable importance in prediction 30-day mortality (variables selected by the stepwise LR analysis).

When using the statistically significant variables selected by the stepwise LR analysis in Table 3, the top five variables with the greatest predictive ability are age, hypertension, dementia, sex, and ASA classification according to the PI method, and CHF, CCI, ASA, age, and RCRI according to the GI method (Figure 4).

**Figure 5:**
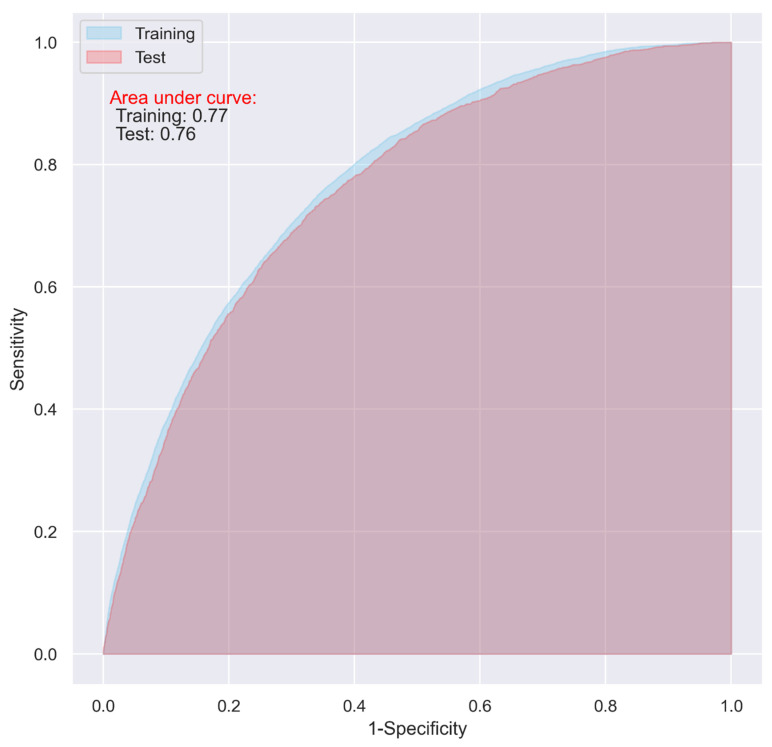
ROC of the CNN model including the variables with a relative importance of >0.05 in the stepwised LR model.

**Figure 6:**
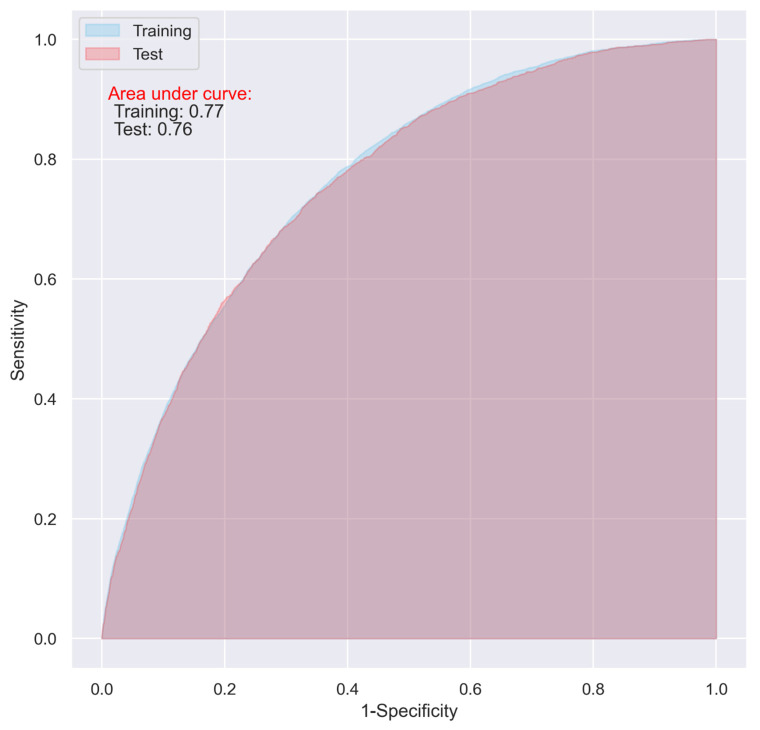
ROC of the LR model including the variables with a relative importance of >0.05 in the stepwised LR model.

Using the variables with a relative importance of >0.05, >0.10, or >0.20 in the stepwise LR model, we reevaluated the predictive ability of the CNN and LR models. It turned out that, even though the overall accuracy of the two models decreased (from 0.74 to 0.71, and 0.73 to 0.70 in the test dataset for the CNN and LR, respectively), the two models using the variables with a relative importance of >0.05 performed best, with an AUC of as high as 0.76 (Table 4), and the overfitting problem in both the models was also significantly reduced (Figure 5 and Figure 6).

## 4. Discussion

Predicting the trajectory of care provided and identifying futile aspects of care, is of paramount importance in clinical practice. For that reason, the use of different risk scores or individual patient characteristics may guide clinicians to better determine how to proceed with care as well as aid in discussions with patients and their relatives, which can avoid the escalation of care with non-futile interventions. To the best of our knowledge, the current study is the largest to date investigating the predictive ability of LR and CNN, and demonstrating the relative importance of the individual variables, using readily available variables in clinical settings to predict 30-day postoperative mortality in hip fracture patients. Both models demonstrated an acceptable predictive ability on the test dataset with an AUC of as high as 0.76 [31]. The CNN model’s performance was not superior to the LR model. This suggests that the LR model is the preferable method for predicting mortality in this patient population as it has the same predictive ability but is simpler to implement in research and clinical practice. The calibration plot for the test dataset reveals that both the models tend to underestimate the probability of 30-day mortality at the lower probability end and overestimate the probability at the higher probability end, and the prediction at the higher probability end appears unstable (Figure S5). The potential reason for this phenomenon deserves further investigation, but it could be partly explained by the small proportion (7.6%) of the patients who died within 30 days postoperatively. Imbalanced data where classes are not represented equally are common in clinical studies. The problem can be tackled in different ways, including changing performance metrics, resampling dataset, data augmentation, adding penalties to the model, etc. There is no rule of thumb for which method should be used, either in isolation or in combination, but can only be determined through a process of trial-and-error. In the current study, we only presented the performance of the training dataset based on the SMOTE; however, its variations such as the majority weighted SMOTE and other oversampling methods or class-weighted training methods deserve further investigation in the future.

The importance of the variables in the prediction were evaluated twice, first with all the variables and thereafter on a pruned list of variables chosen using bidirectional stepwise variable selection, which removed variables that were statistically non-significant in the LR analysis. However, a difference was observed for the 5 most important predictive variables when comparing the results of the PI and GI. The PI, based on LR model, indicates that some simple variables, such as age, hypertension, dementia, and sex are important; while Gini importance indicates that some composite variables, such as ASA classification, CCI, and RCRI have a higher predictive ability.

This result is consistent with a previous systematic review that found no benefit of machine learning, including neural networks, over logistic regression for clinical prediction models [34]. Nevertheless, several smaller studies have found different results when comparing regression models to neural networks to predict 1-year mortality after hip fracture surgery [35,36,37,38]. Two of the studies compared logistic regression with a neural network and found that the neural network outperformed the alternative [35,36]. These studies were, however, limited by small datasets comprising 286 and 434 cases, respectively [35,36]. Another study investigated the same question using a larger dataset with 2150 patients and also found that a neural network was better than a logistic regression model for predicting mortality [37]. Finally, the largest of these studies, containing 10,534 patients, found that a neural network outperforms a Cox proportional hazards model [38]. While the small sample size might limit the generalizability of the neural networks produced in the first two studies [39], the fact remains that all the studies calculated significantly larger AUCs for their neural networks compared to our CNN, with most having an AUC > 0.9 [35,36,37,38]. Despite all of our efforts, such as using different split partitions in the training and test datasets, introducing dropout layers, and including only variables with relative higher importance, to improve the performance of the CNN model, the AUC did not reflect the same high values as previous studies. This may in part be due to the inclusion of variables that were not available in the current study, such as mobility, functional status, socioeconomic characteristics, hospital level, surgeon volume, and hospital volume. Although these variables could theoretically increase the predictive ability of the models, these are not very useful or readily available in clinical practice. Additionally, to be noted, is that the previous studies focused mainly on 1-year mortality rather than 30-day postoperative mortality. However, including only variables with relatively higher importance seems an effective method for reducing overfitting and increasing the performance in the current study cohort.

Threshold-dependent metrics are widely used to evaluate predictive models’ performance in the clinical settings, often on the grounds that binary predictions are necessary for treatment planning. They may help to identify a model that best predicts a specific outcome, assess the importance of a variable in prediction, and suggest the direction for model improvement. However, they show a reduced discrimination ability in cases where more than two occurrence probabilities exist [27]. Moreover, the threshold *τ* used in the current study was identified by maximizing the Youden index (or sum of sensitivity + specificity − 1). However, in cases where there are wide disparities in the cost of false negatives vs. false positives, it may be critical to minimize one type of classification error. For example, in a cancer screening test, sensitivity is more important because a delay in the diagnosis might result in the need for more expensive treatments and increase the risk of fatal outcomes. In this case, the maximized value of the Youden index would not be a useful threshold for this type of classification, and we would expect to achieve a higher sensitivity and prioritize minimizing false positives during the classification. On the other hand, threshold-independence has been regarded as a desirable property of the performance metrics such as AUC, however, they are limited to measuring discrimination ability (the ability to distinguish presence above absence) and cannot measure calibration. Therefore, rather than just reporting the AUC, the complete ROC curve is recommended to be reported together with the AUC [30].

Given the simplicity in clinical application of LR, and that no significant difference in predictive ability was observed between the LR and CNN models in our data, a LR model using age, hypertension, dementia, sex, and ASA classification would be useful to predict the acute mortality in traumatic hip fracture patients after surgery. Therefore, the PI will be used instead of the GI to compare the predictive power for individual variables in the current study.

Several indices have been proposed in order to predict postoperative mortality after hip fracture surgery, such as the Nottingham Hip Fracture Score (NHFS), CCI, and Physiological and Operative Severity Score for the enumeration of Mortality and morbidity (POSSUM) [16,40,41,42]. The association between the CCI and postoperative mortality has been observed in numerous studies [7,8,9,38,43,44]. The ability of the CCI to predict postoperative mortality in hip fracture patients has also been compared to the NHFS and POSSUM in prior studies [45,46]. In some, the NHFS performs slightly better and in others POSSUM performs marginally worse, but overall, these indices have a comparable predictive ability when all studies are taken into consideration [45,47,48]. In general, all three indices demonstrate AUCs tending to lie between 0.7 and 0.8 depending on the study.

As can be seen by the PI, the RCRI presented a similar predictive ability to the CCI in the unfiltered variable set and even outperformed it in the feature-reduced models. This seems to indicate that the RCRI has the same level of predictive ability as the CCI. Consequently, the RCRI should also be comparable to the NHFS and POSSUM as they both have an equivalent predictive power to the CCI [45,46]. It might therefore be argued that the RCRI is preferable to all of these indices. The RCRI makes use of six binary variables, all of which can be retrieved without further blood tests or intraoperative data [17]. Conversely, all the other indices require significantly more variables [16,40,41,42]. The NHFS itself necessitates the calculation of a completely different score, the Abbreviated Mental Test score, in order to function [40]. Both the NHFS and POSSUM also call for blood tests, in addition to the use of vital signs and intraoperative data already required by POSSUM [40,41,42].

The importance of applying risk stratification tools such as the RCRI for patients undergoing noncardiac surgery is stressed by The American College of Cardiology’s and American Heart Association’s (ACC/AHA) most recent guidelines [49]. In clinical practice, they recommend initiation of beta-blockers in patients with an RCRI ≥ 3 to reduce the risk of adverse postoperative cardiac events. This association has been previously shown in patients undergoing noncardiac surgery [18].

Nevertheless, the fact remains that irrespective of which risk score is used, none seems to achieve anything beyond an acceptable predictive ability based on the AUC [31]. POSSUM and NHFS scores are poorly calibrated in hip fracture patients, often resulting in an underestimation of mortality in the lower risk strata and overestimating mortality in the higher risk strata and the CCI does not appear to fare any better [45,50]. The simplest explanation may be that all of these indices, including the RCRI, are missing factors required to paint a complete picture. Each of these scoring systems are in essence measures of a patient’s comorbidity burden; most do not include any measures of functional status of the patient prior to surgery [16,40,41,42]. The solution may therefore be the use of several indices in order to capture all aspects of a patient’s preoperative risk, for example a comorbidity index, a frailty index, which would include mobility and functional status, and a fitness-for-surgery index, such as the ASA classification [51,52]. This is in part supported by results presented in the current study, where the ASA classification was retained in addition to the RCRI and CCI after removing variables that demonstrated a high degree of collinearity; this indicates that the ASA classification is providing new information to the model that is not already found in the other indices.

A central goal of developing the CNN and LR models is to facilitate clinical decision-making, for example by predicting 30-day postoperative mortality. In these situations, however, preoperative information and time are often limited, which makes distinguishing the specific variables with the highest predictive ability essential. Being able to predict outcomes allows clinicians to make optimal and timely decisions regarding interventions as well as allows for better discussions with patients and their relatives regarding medical and surgical strategies. To be able to achieve this, variables that result in models with both high sensitivity and high specificity are fundamental.

This study benefits from a large dataset based on ten consecutive years of data from the Swedish National Quality Registry for Hip Fracture Patients. Contributions to this database from all orthopedic departments in Sweden allows for high case coverage, ranging between 80–90% [6,53]. Nonetheless, due to the nature of register data, the models were limited to the variables available in the national quality registers. Future analyses including factors such as functional status and frailty would be of value. This study also limits its scope to the prediction of 30-day postoperative mortality. Consequently, additional studies investigating long-term mortality as well as functional outcome and complications should be conducted.

## 5. Conclusions

The CNN and LR models, both achieved an acceptable performance in terms of AUC in identifying patients at risk of mortality 30 days after hip fracture surgery. The overfitting issue presented in the full CNN or LR models might be mitigated by including only the variables with a higher importance. The most important variables for prediction, based on the variables used in the current study are age, hypertension, dementia, sex, ASA classification, and RCRI.

## Figures and Tables

**Table 1:** Characteristics of the traumatic hip fracture patients in Sweden between 2008 and 2017.

Variable	Total (*n* = 134,915)	Alive (*n* = 124,707)	Dead (*n* = 10,208)	*p*-Value
Age, mean ± SD	82.0 ± 10.0	81.5 ± 10.1	86.9 ± 7.3	<0.001
Male * (%)	42,988 (31.9)	38,314 (30.7)	4674 (45.8)	<0.001
ASA classification * (%)				<0.001
1	6656 (5.0)	6536 (5.34)	120 (5.03)	
2	48,264 (36.4)	46,507 (38.0)	1757 (17.6)	
3	66,857 (50.5)	60,857 (49.7)	6000 (60.0)	
4	10,534 (8.0)	8459 (6.9)	2075 (20.7)	
5	135 (0.1)	79 (0.1)	56 (0.1)	
RCRI (%)				<0.001
0	79,941 (59.3)	75,864 (60.8)	4077 (39.9)	
1	36,848 (27.3)	33,476 (26.8)	3372 (33.0)	
2	13,086 (9.7)	11,284 (9.1)	1802 (17.7)	
3	3971 (2.9)	3245 (2.6)	726 (7.11)	
≥4	1069 (0.8)	838 (0.7)	231 (2.26)	
Fracture type * (%)				<0.001
Non-displaced cervical (Garden 1–2)	17,868 (13.3)	16,840 (13.5)	1028 (10.1)	
Displaced cervical (Garden 3–4)	50,172 (37.2)	46,248 (37.1)	3924 (38.5)	
Basicervical	4480 (3.3)	4126 (3.3)	354 (3.5)	
Pertrochanteric (two fragments)	26,859 (19.9)	24,775 (19.9)	2084 (20.4)	
Pertrochanteric (multiple fragments)	24,493 (19.2)	22,487 (18.0)	2006 (19.7)	
Subtrochanteric	10,988 (8.2)	10,178 (8.2)	810 (7.9)	
Surgery type * (%)				<0.001
Pins or screws	23,548 (17.4)	21,849 (17.5)	1609 (15.8)	
Screws or pins with sideplate	34,902 (25.9)	32,146 (25.8)	2756 (27.0)	
Intramedullary nail	31,992 (23.7)	29,496 (23.7)	2496 (24.5)	
Hemiarthroplasty	34,596 (25.7)	31,473 (25.3)	3123 (30.6)	
Total hip replacement	9889 (7.33)	9676 (7.8)	213 (2.1)	
CCI (%)				<0.001
≤4	59,611 (44.2)	57,634 (46.2)	1977 (19.4)	
5–6	50,247 (37.2)	45,733 (36.7)	4514 (44.2)	
≥7	25,057 (18.6)	21,340 (17.1)	3717 (36.4)	
PVD (%)	5890 (4.4)	5236 (4.2)	654 (6.4)	<0.001
COPD (%)	15,577 (11.6)	13,933 (11.2)	1644 (16.1)	<0.001
Liver disease (%)	1370 (1.0)	1232 (1.0)	138 (1.4)	<0.001
Dementia (%)	27,304 (20.2)	27,789 (19.1)	3515 (34.4)	<0.001
Connective tissue disease (%)	6487 (4.8)	6036 (4.8)	451 (4.4)	0.055
Cancer (%)	14,560 (10.8)	13,108 (10.5)	1452 (14.2)	<0.001
Metastatic carcinoma (%)	2962 (2.2)	2498 (2.0)	464 (4.6)	<0.001
MI (%)	8063 (5.9)	6789 (5.4)	1274 (12.5)	<0.001
CHF (%)	21,097 (15.6)	17,475 (14.0)	3633 (35.8)	<0.001
Hypertension (%)	51,756 (38.4)	47,990 (38.5)	3766 (36.9)	0.001
Arrhythmia (%)	24,998 (18.5)	22,305 (17.9)	2693 (26.4)	<0.001
CeVD (%)	23,382 (17.3)	21,036 (16.9)	2346 (23.0)	<0.001
Peptic ulcer disease (%)	4328 (3.21)	3918 (3.1)	410 (4.0)	<0.001
Diabetes (%)	19,856 (14.7)	18,166 (14.6)	1690 (16.6)	<0.001
Hemiplegia (%)	2911 (2.2)	2715 (2.2)	196 (1.9)	0.086
Chronic kidney disease (%)	6945 (5.2)	5774 (4.6)	1171 (11.5)	<0.001

* Listwise deletion was used for the observations with missing values.

**Table 2:** Odds ratio (OR) of predictor variables for 30-day mortality after surgery (full LR model).

Variable	OR	*p*-Value	95% Confidence Interval	
			Upper Limit	Lower Limit
RCRI				
0	Reference			
1	1.371	0.210	0.837	2.246
2	1.787	0.247	0.669	4.774
3	2.249	0.280	0.516	9.802
≥4	2.764	0.324	0.367	20.821
Age	1.074	<0.001	1.070	1.078
Male	1.927	<0.001	1.842	2.017
PVD	1.146	0.005	1.042	1.259
COPD	1.291	<0.001	1.209	1.378
Liver disease	2.150	<0.001	1.766	2.618
Dementia	1.837	<0.001	1.738	1.941
Connective tissue disease	0.948	0.320	0.852	1.054
Cancer	1.166	<0.001	1.076	1.262
Metastatic carcinoma	2.751	<0.001	2.400	3.152
ASA classification				
1	Reference			
2	1.271	0.013	1.051	1.536
3	2.149	<0.001	1.782	2.593
4	4.228	<0.001	3.484	5.131
5	12.007	<0.001	7.928	18.185
Fracture type				
Non-displaced cervical (Garden 1–2)	Reference			
Displaced cervical (Garden 3–4)	1.367	<0.001	1.240	1.506
Basicervical	1.266	0.008	1.064	1.506
Pertrochanteric (two fragments)	1.337	<0.001	1.141	1.568
Pertrochanteric (multiple fragments)	1.443	<0.001	1.226	1.699
Subtrochanteric	1.455	<0.001	1.222	1.731
Surgery type				
Pins or screws	Reference			
Screws or pins with sideplate	0.947	0.468	0.817	1.098
Intramedullary nail	0.895	0.168	0.765	1.048
Hemiarthroplasty	0.998	0.957	0.915	1.088
Total hip replacement	0.593	<0.001	0.505	0.696
CCI				
≤4	Reference			
5–6	1.150	<0.001	1.064	1.242
≥7	1.143	0.037	1.008	1.295
MI	1.070	0.786	0.654	1.752
CHF	1.472	0.124	0.899	2.410
Hypertension	0.649	<0.001	0.618	0.682
Arrhythmia	0.930	0.010	0.881	0.983
CeVD	0.854	0.528	0.522	1.396
Peptic ulcer disease	1.038	0.521	0.927	1.162
Diabetes	0.808	0.396	0.494	1.322
Hemiplegia	0.8620	0.073	0.732	1.014
Chronic kidney disease	1.126	0.638	0.687	1.844

**Table 3:** Odds ratio (OR) of predictor variables for 30-day mortality after surgery (stepwise LR model).

Variable	OR	*p*-Value	95% Confidence Interval	
			Upper Limit	Lower Limit
RCRI				
0	Reference			
1	1.500	0.000	1.373	1.638
2	2.138	0.000	1.857	2.462
3	2.948	0.000	2.436	3.569
≥4	4.011	0.000	3.105	5.182
Age	1.074	0.000	1.070	1.078
Male	1.933	0.000	1.847	2.022
PVD	1.147	0.004	1.043	1.261
COPD	1.289	0.000	1.208	1.376
Liver disease	2.161	0.000	1.776	2.630
Dementia	1.838	0.000	1.741	1.942
arrythmia	0.931	0.011	0.881	0.983
Cancer	1.165	0.000	1.077	1.261
Metastatic carcinoma	2.750	0.000	2.405	3.145
ASA class				
1	Reference			
2	1.270	0.014	1.051	1.536
3	2.148	0.000	1.781	2.591
4	4.229	0.000	3.485	5.131
5	11.987	0.000	7.918	18.147
Displaced cervical (Garden 3–4)	1.366	0.000	1.267	1.473
Basicervical	1.217	0.004	1.064	1.391
Peritrochanteric (two fragments)	1.271	0.000	1.169	1.382
Peritrochanteric (multiple fragments)	1.373	0.000	1.251	1.506
Subtrochanteric	1.384	0.000	1.235	1.552
Diabetes	0.739	0.000	0.680	0.802
Intramedullary nail	0.944	0.094	0.883	1.010
CeVD	0.780	0.000	0.722	0.843
Total hip replacement	0.595	0.000	0.515	0.687
CCI				
≤4	Reference			
5–6	1.147	0.000	1.063	1.238
≥7	1.143	0.028	1.014	1.288
Hemiplegia	0.861	0.070	0.732	1.013
CHF	1.345	0.000	1.237	1.463
Hypertension	0.649	0.000	0.618	0.682

**Table 4:** Predictive ability of the CNN, LR, and RF models.

Model	Training				Test			
	Accuracy	Specificity	Sensitivity	AUC	Accuracy	Specificity	Sensitivity	AUC
CNN ^1^	0.77	0.76	0.80	0.87	0.74	0.76	0.52	0.72
CNN ^2^	0.80	0.79	0.80	0.89	0.76	0.79	0.46	0.71
CNN ^3^	0.79	0.78	0.80	0.88	0.75	0.77	0.47	0.69
CNN ^4^	0.70	0.71	0.70	0.77	0.71	0.71	0.68	0.76
LR	0.74	0.74	0.74	0.83	0.73	0.75	0.57	0.73
LR ^5^	0.74	0.72	0.76	0.82	0.72	0.73	0.60	0.74
LR ^6^	0.74	0.74	0.74	0.83	0.73	0.75	0.58	0.73
LR ^7^	0.74	0.74	0.75	0.83	0.73	0.74	0.58	0.73
LR ^4^	0.70	0.69	0.71	0.76	0.70	0.70	0.69	0.76
RF	0.68	0.54	0.81	0.76	0.57	0.55	0.76	0.72

^1^ The training and test datasets were split into 80%:20% partitions. ^2^ The training and test datasets were split into 85%:15% partitions. ^3^ The training and test datasets were split into 90%:10% partitions. ^4^ Using the variables with a relative importance of >0.05 in the stepwised LR model. ^5^ Including an elastic net penalty. ^6^ Including an L1 penalty. ^7^ Including an L2 penalty.

## Data Availability

The ethical approval obtained for this study prevents the human data being shared publicly to protect patients’ privacy. Interested readers can contact Shahin Mohseni with their research plan to request access. This would be passed to Rikshöft who will decide whether they can access the data directly from the relevant Swedish authority.

## Supplementary Materials

The following are available online at https://www.mdpi.com/article/10.3390/jpm11050353/s1, Figure S1: Structure of the convolutional neural network, Figure S2: Training and test of the machine learning models; Figure S3: Performance of the CNN model with different number of filters; Figure S4: Performance of the CNN model with different number of epochs; Figure S5: Calibration curve of the CNN and LR models on the test dataset.
