# Supplementary material for: Predictive Values of Preoperative Characteristics for 30-Day Mortality in Traumatic Hip Fracture Patients
Source: J Pers Med. 2021 Apr 28;11(5):353. doi: 10.3390/jpm11050353 (PMC8146802; doi:10.3390/jpm11050353)
Supplement: Supplementary file 1 [file jpm-11-00353-s001.zip › jpm-1149058-supplementary.pdf]

Journal of
Personalized Medicine

Supplementary materials

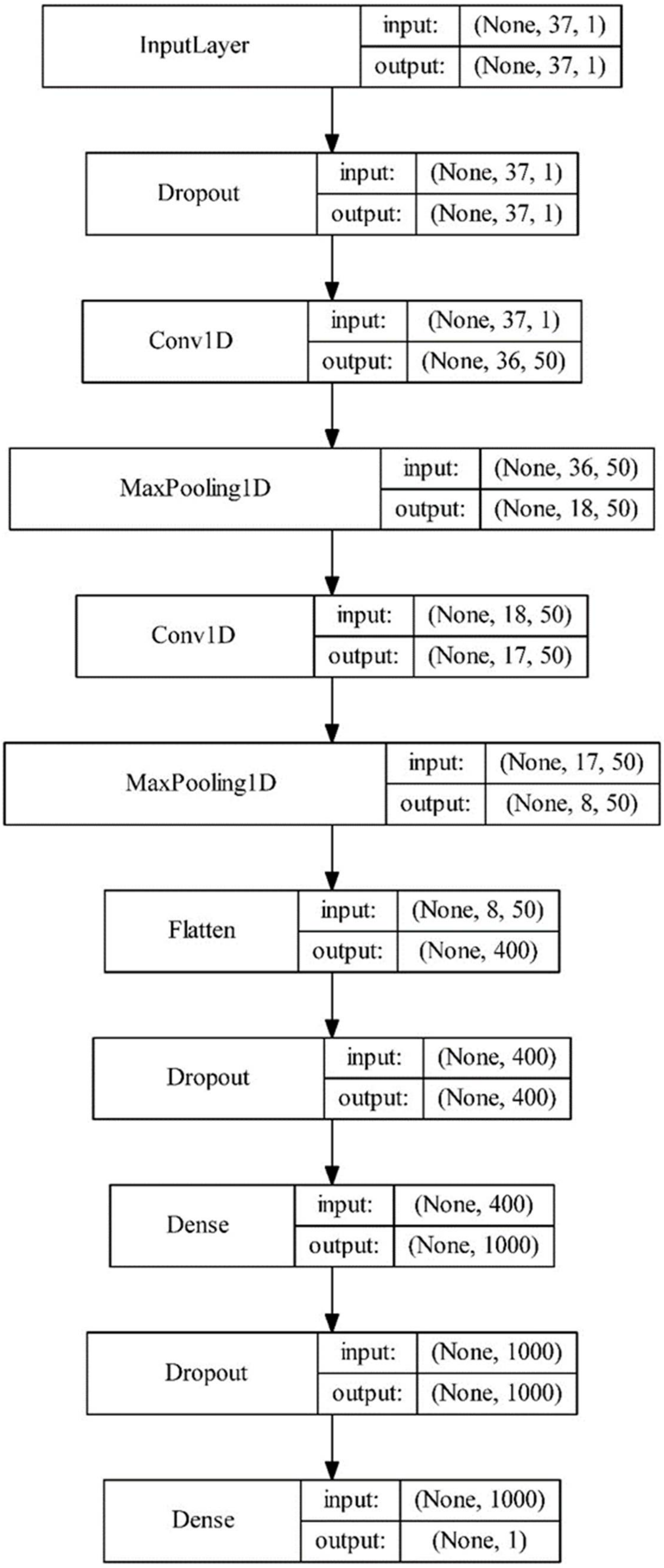

Figure S1. Structure of the convolutional neural network.

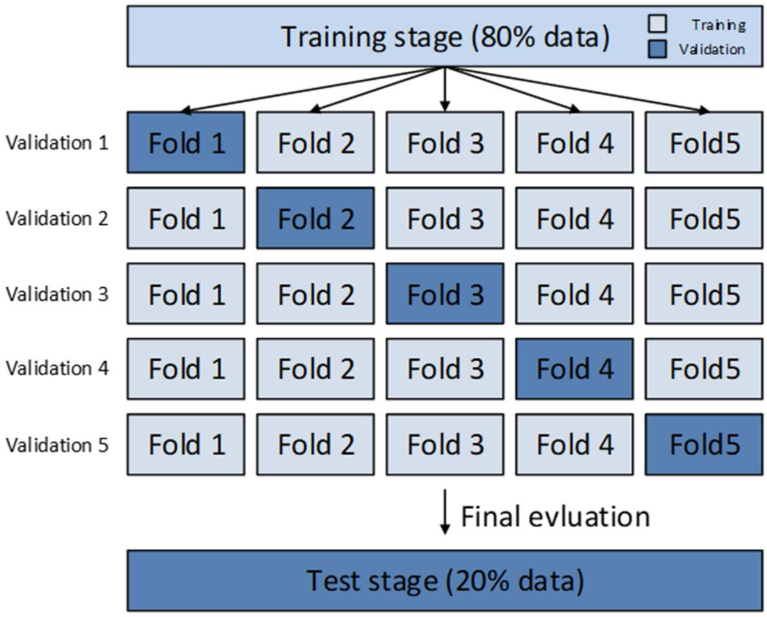

Figure S2. Training and test of the machine learning models.

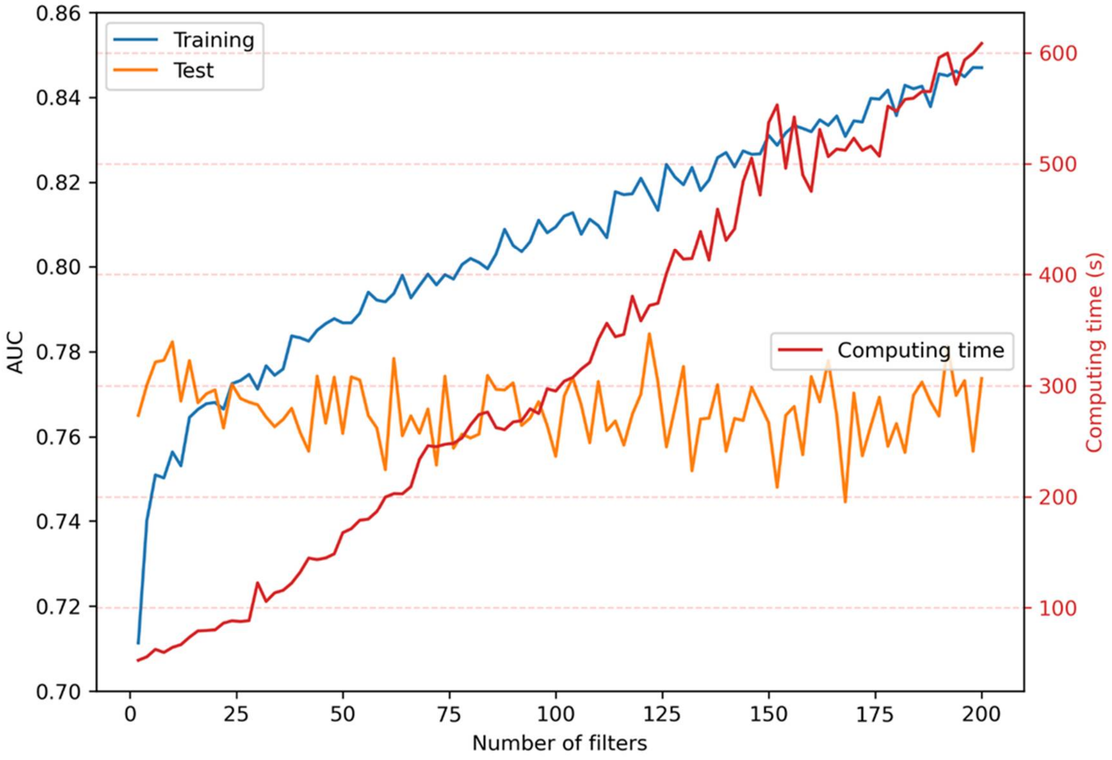

Figure S3. Performance of the CNN model with different number of filters.

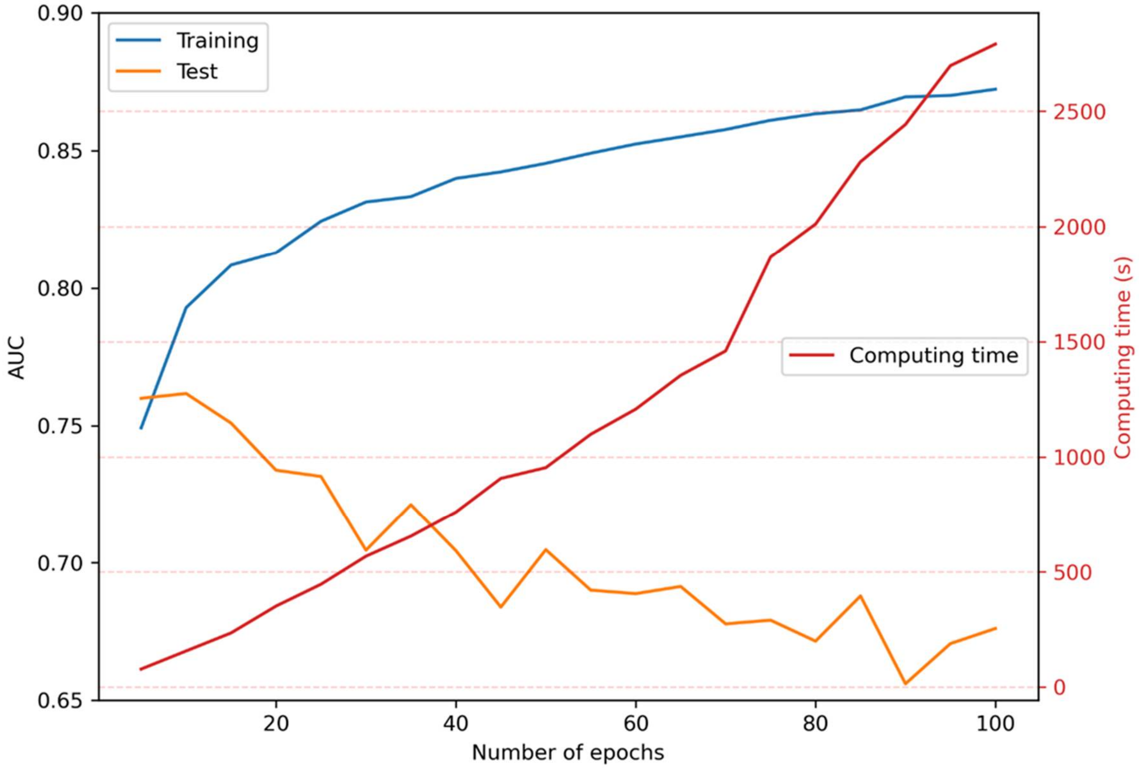

Figure S4. Performance of the CNN model with different number of epochs.

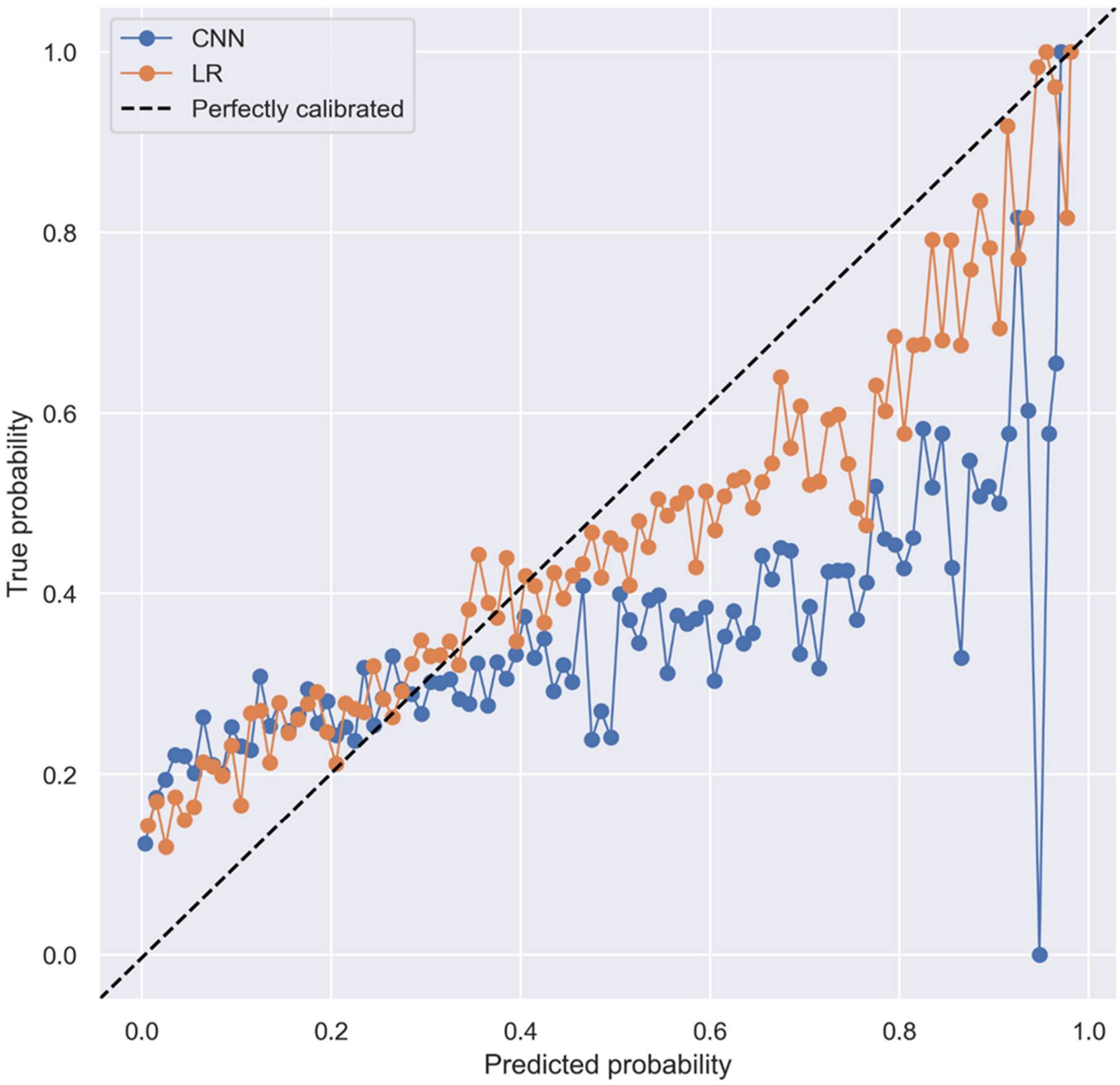

Figure S5. Calibration curve of the CNN and LR models on the test dataset.
